# Predicted structure of a Minus-C OBP from *Batocera horsfieldi* (Hope) suggests an intermediate structure in evolution of OBPs

**DOI:** 10.1038/srep33981

**Published:** 2016-09-23

**Authors:** Zhi-Chuan Zheng, Dong-Zhen Li, Aiming Zhou, Shan-Cheng Yi, Hao Liu, Man-Qun Wang

**Affiliations:** 1 Hubei Insect Resources Utilization and Sustainable Pest Management Key Laboratory, College of Plant Science and Technology, Huazhong Agricultural University, Wuhan 430070, P. R. China

## Abstract

Odorant binding proteins (OBPs) transport hydrophobic odorants from the environment to odorant receptors and play an important role in specific recognition of volatiles. Here, we expressed and purified a minus-C OBP, BhorOBPm2, from *Batocera horsfieldi*, a major pest of *Popolus*, to determine its binding characteristics with 58 candidate volatiles using a fluorescence competition-binding assay. We showed that BhorOBPm2 exhibited high binding affinity with chain volatiles and that ligands were selected based on chain length. In order to elucidate the binding mechanism, homology modeling and molecular-docking experiments were performed to investigate interactions between BhorOBPm2 and volatiles. The predicted structure with only two disulfide bonds showed one continuous channel for ligand binding, similar to classic OBPs AgamOBP1 and CquiOBP1. Unexpectedly, we observed a larger binding pocket for BhorOBPm2 and broader specificity for ligands than classic OBPs due to the expansive flexibility of BhorOBPm2 resulting from a lack of disulfide bonds. These findings suggested that BhorOBPm2 might present an intermediate structure in the evolution of OBPs. Furthermore, we designed two mutant proteins to simulate and verify functions of the C-terminal region. The changes in binding affinity observed here indicated a novel action differing from that of the “lid” described in previous studies.

The olfactory system is capable of detecting and distinguishing thousands of environmental volatiles that play a key role in behaviors that include foraging, host-seeking, mating, and oviposition^1^,^2^,^3^. Olfaction in insects depends upon the antennae, the principal olfactory organ of insects, which has olfactory sensory neurons housed in the sensilla^4^. Odorant binding proteins (OBPs) are highly expressed in the sensillum lymph and can convey odorant molecules through the sensillum lymph to odorant receptors on the membranes of olfactory sensory neurons^5^,^6^,^7^. OBPs that bind and convey signals from pheromones are called pheromone-binding proteins (PBPs), while those that convey signals from general odorants are called general odorant-binding proteins (GOBPs)^8^,^9^.

Although genome and transcriptome analyses have defined a large number of OBP genes^10^,^11^,^12^, the crystal structures of insect OBPs are rarely reported. Among the resolved OBP structures, the classic OBPs *Anopheles gambiae* AgamOBP1, *Culex quinquefasciatus* CquiOBP1, and *Aedes aegypti* AaegOBP1 have a high degree of sequence identity and similar structure^13^,^14^,^15^. As with observed structures of other OBPs, they have three disulfide bridges stabilizing the three-dimensional (3D) structure and constituting an internal pocket for binding ligands via a six-α-helix fold. The distinctive feature of these OBPs is the unique binding pocket with a long hydrophobic tunnel capable of binding the long-chain ligand, followed by dimer formation. The binding pocket of each monomer has an opening created by helices, allowing the ligand molecule to enter into the binding pocket. Additionally, the surface of the binding pocket in these OBPs is largely hydrophobic. Studies show the interactions of PEG-AgamOBP1 and MOP-CquiOBP1 were only hydrophobic, in contrast to *Bombyx mori* BmorGOBP2 with the ligand bombykol, and *Drosophila melanogaster* LUSH with the ligand alcohol^16^,^17^. All the common features of the three OBPs suggest that the ligands with a long hydrophobic chain fit better in the binding pocket. Furthermore, AgamOBP1, CquiOBP1 and AaegOBP1 also have the similar ligand-release mechanism in a pH-dependent fashion. The C-terminal region of these OBPs forms a wall-like “lid” over the binding pocket, which moves away from the binding pocket by disrupting hydrogen bonds at lower pH.

Other non-classical OBPs were also identified, including minus-C OBPs, which contain only four conserved cysteines^18^, and plus-C OBPs, which contain more than six conserved cysteines^19^. These were often separated from classical OBPs and into different families to facilitate phylogenetic analysis in order to promote functional studies of OBPs at the molecular level^12^. Unfortunately, crystal structures of non-classical OBPs are rarely reported. *Apis mellifera* AmelOBP14 was the first and only 3D structure of a minus-C OBP, and was characterized by only two disulfide bonds^20^. Aside from AmelOBP14 structures in complex with N-phenyl-1-naphthylamine (1-NPN), eugenol and citralva were investigated in order to attempt to explain their strong binding activity. The results revealed a third disulfide bridge that did not disturb the AmelOBP14 fold^20^. The new disulfide bridge caused constricted flexibility, impacting the ability to adapt its binding pocket to fit different odorants having various functional groups^21^. Combined with earlier studies of the origins and evolutionary history of the chemosensory system^22^, it was suggested that minus-C OBPs might be ancestral proteins, and that the OBP driving force in evolution concerns the introduction of a larger number of disulfide bridges and additional complexity^20^.

However, due to the lack of research on minus-C OBPs, little information regarding sequence-specific relationships between minus-C and classical OBPs are available. Studies addressing binding affinities and structural characteristics associated with minus-C OBPs and their ligands could contribute substantial information as compared with sequence studies. Recently, molecular-docking simulations involving OBP homology models provide reliable and easier approaches for studying OBP structures^23^,^24^,^25^,^26^,^27^.

*Batocera horsfieldi* (Hope) (Coleoptera: Cerambycidae) is widely distributed in China and is an important pest of the *Populus* (Salicales: Salicaceae) species^28^. The larvae bore into the inner bark and the trunk, resulting in wind damage, and *B. horsfieldi* adults emerge in May in China to feed mainly on the branches of *Rosa multiflora* Thunb (Rosales: Rosaceae). After mating, the females travel back to *Populus* for oviposition under the bark of trees^29^,^30^,^31^. *B. horsfieldi* has a sensitive olfactory system that is essential for host location and mating, and a number of olfactory-related genes have been identified in *B. horsfieldi*^32^,^33^,^34^. Here, we cloned the cDNA of minus-C OBP2 (BhorOBPm2) from *B. horsfieldi* and expressed and purified the protein *in vitro*. Fluorescence-binding assays showed that BhorOBPm2 recognized ligands based on chain length, and homology modeling and molecular-docking analysis indicated that BhorOBPm2 shared similar characteristics with some classic OBPs, including AgamOBP1, CquiOBP1, and AaegOBP1, but not with the minus-C OBP AmelOBP14. Additionally, our results showed that BhorOBPm2 exhibits intermediate structural features between minus-C OBPs and these classical OBPs, implying that this minus-C OBP might be an ancestral protein of these classic OBPs. Furthermore, BhorOBPm2 exhibited low binding affinity at low pH and variable affinities associated with C-terminal mutants, which was not consistent with the “lid” function of AaegOBP1.

## Results

### BhorOBPm2 cloning and sequence analysis

BhorOBPm2 was obtained from the cDNA library, and using gene-specific primers, a full-length cDNA encoding BhorOBPm2 was cloned. Sequence analysis showed that the full open reading frame contained 435 bp encoding 145 amino acid residues, with a predicted MW of 14.87 kDa. For BhorOBPm2, SignalP (http://www.cbs.dtu.dk/services/SignalP/) predicted a 21-amino acid signal peptide, and ExPASy (http://web.expasy.org/compute_pi/) predicted an isoelectric point of 5.28. The alignment of the BhorOBPm2 amino acid sequence with those of corresponding OBPs from other species was conducted^33^, revealing that BhorOBPm2 had only four cysteine residues and belonged to the minus-C insect OBP sub-family according to the following pattern: X42-C1-X30-C2-X37-C3-X19-C4-X12 (X denotes any amino acid).

### BhorOBPm2-ligand characteristics and fluorescence-binding assays

**Figure 1:**
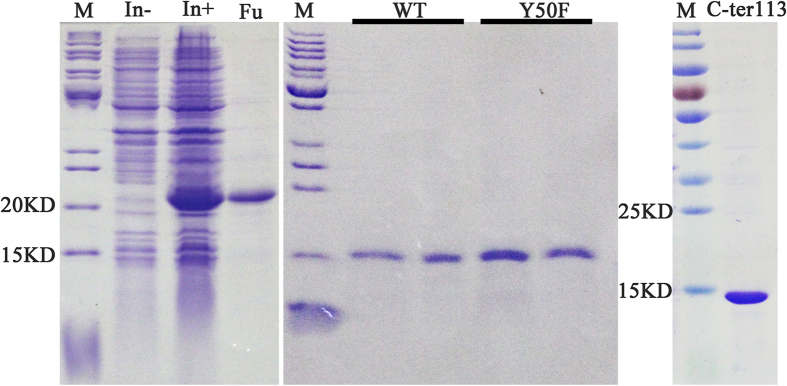
SDS-PAGE analyses showing the expression and purification of BhorOBPm2, as well as two mutants. M, molecular marker; In- and In+, bacterial cells before and after induction by IPTG, respectively; Fu, purified fusion protein BhorOBPm2-WT. The last two pictures show the purified protein of BhorOBPm2-WT, Y50F and C-ter113.

**Figure 2:**
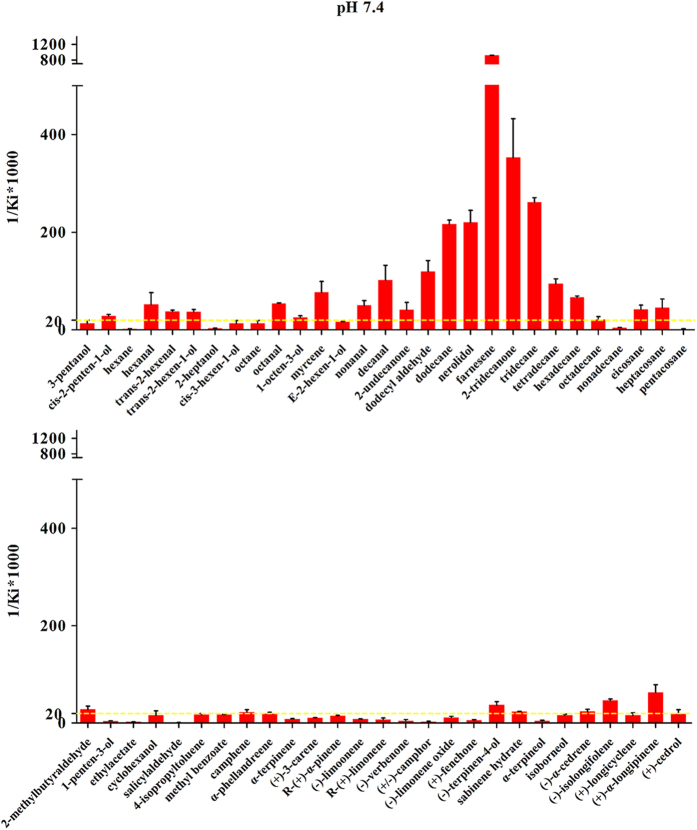
Binding affinities (indicated by 1/Ki*1000) of various ligands to BhorOBPm2-WT at pH 7.4. The first picture shows the binding ability of ligands with long chain. The second picture shows the binding ability of ligands without long chain.

The purified proteins were confirmed by SDS-PAGE (Fig. 1) and evaluated by fluorescence-binding assays using 58 ligands to investigate the ligand-binding mechanisms (Supplementary Table S1). Ligands were divided into two major groups based on shape (Supplementary Fig. S1): ligands with or without long chains. Plane structures of the ligands are shown in Supplementary Fig. S1. The wild-type BhorOBPm2 binding affinities (indicated by 1/K_i_ × 1000) with ligands are shown in Supplementary Table S2. By comparing binding values between groups 1 and 2, we determined that ligands with long carbon chains (group 1) exhibited a higher binding affinity as compared with the ligands from group 2 at pH 7.4 (Fig. 2). Additionally, we observed that overall binding activity was dependent upon chain length. Farnesene, with a backbone of 12 carbon atoms, exhibited the strongest binding affinity (1/K_i_(μM) × 1000 = 1159.11) at pH 7.4. Additionally, carbon chains of length 6 to 12 showed increased affinities, while those with chain lengths of 12 to 16 carbons exhibited decreasing binding affinities (Fig. 2). Furthermore, most of the ligands exhibited a lower binding ability at pH 5.0 as compared with those observed at pH 7.4 (Supplementary Table S2). These results suggested that wild-type BhorOBPm2 was capable of selecting the appropriate ligands dependent upon the chain length.

### Molecular modeling and docking

Using SWISS-MODEL, four homologous proteins, including *Tenebrio molitor* THP12 (1C3Y; 35.56%), *Anopheles gambiae* AgamOBP1 (2ERB; 34.86%), *Culex quinquefasciatus* CquiOBP1 (3OGN; 32.11%), and *Aedes aegypti* AaegOBP1 (3K1E; 31.19%), were obtained using a sequence-similarity cut off of 30%.

2ERB was selected as the template for homology modeling, because 2ERB, 3OGN, and 3K1E have homogeneous structures and belong to the same family of insect OBPs. However, it has not been confirmed whether 1C3Y belongs to the same family of OBPs, but it was proposed that 1C3Y is not a proper template for AmelOBP14^20^. More importantly, binding assays showed that the catenulate ligands exhibited better binding affinities than those without chains. This finding agreed with our observations of 2ERB, 3OGN, and 3K1E, which have continuous hydrophobic channels capable of binding long-chain ligands. Additionally, 2ERB exhibited greater sequence similarity with BhorOBPm2 as compared with 3OGN and 3K1E.

Based on stereochemical optimization and energy minimization performed with MOE, a first-rank model with the minimum energy among the 2500 intermediate models was evaluated with the stereochemical quality evaluation tool in MOE (Protein Geometry). A pairwise RMSD of α-carbons between 2ERB and BhorOBPm2 was 1.12 Å (Fig. S2). As shown in Supplementary Fig. S3, all residues were located in the allowed regions according to the Ramachandran map, along with other positive results associated with stereochemical indices (including bond lengths, bond angles, and dihedrals), indicating that the overall stereochemical quality was generally reliable and acceptable.

### Binding pocket

**Figure 3:**
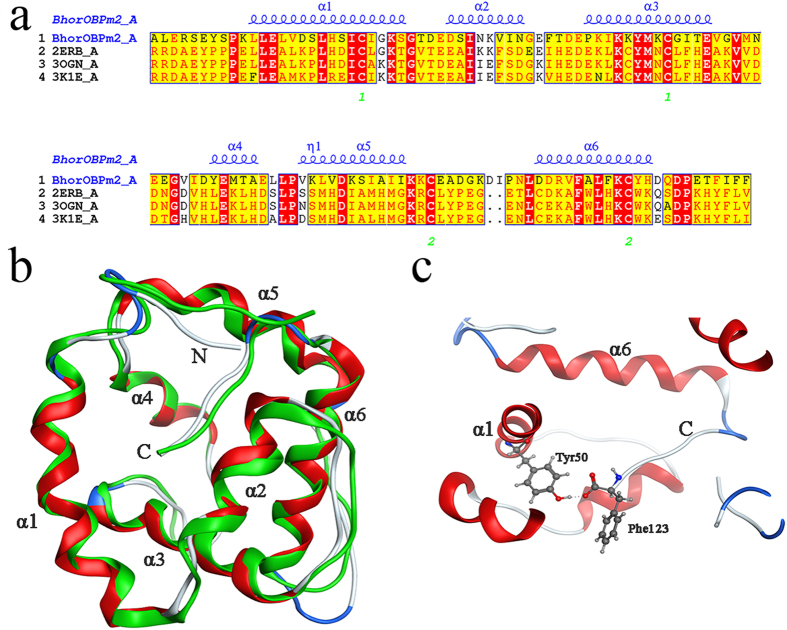
Structure of BhorOBPm2. **(a)** Sequence alignment of BhorOBPm2 and homologous proteins. 2ERB was the template of BhorOBPm2. α-helices are displayed as squiggles. Identical residues are displayed in white on a red background. The disulfide bridges are numbered 1–2 below the sequences. **(b)** Superimposed structures of BhorOBPm2 and the template 2ERB. The model of BhorOBPm2 and crystal structure of 2ERB are shown in red and green, respectively. **(c)** Hydrogen bonding between the hydroxyl of Tyr-50 and the C-terminal carboxylate of Phe-123 from BhorOBPm2. The red atom is oxygen atom. The blue atom is nitrogen-atom. Hydrogen bonds is shown as gray dotted lines.

**Figure 4:**
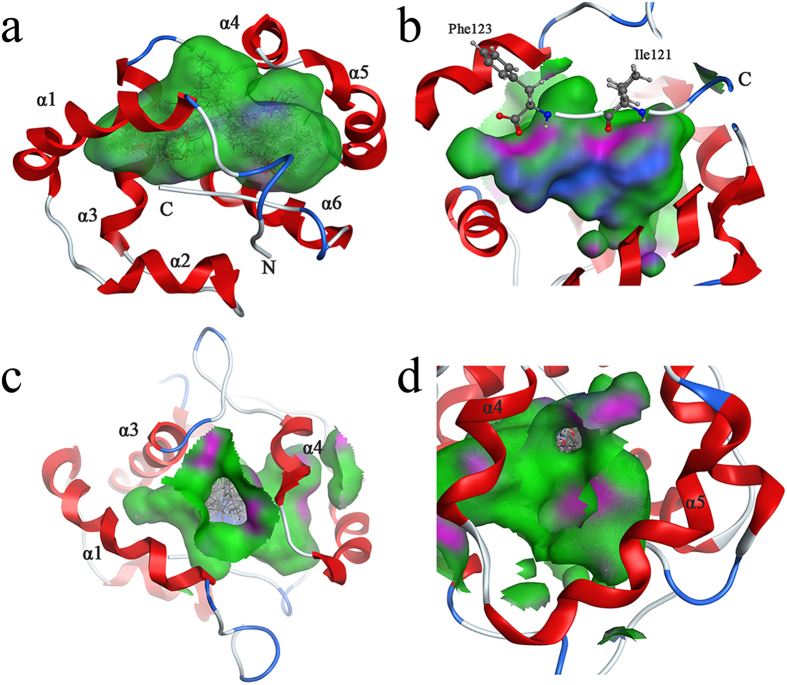
The binding pocket and docking results of BhorOBPm2. The green area expresses hydrophobicity and red areas expresses hydrophilia. (**a**) The binding pocket in BhorOBPm2 core. (**b**) The area of the C-terminal wall. The red atom is oxygen atom. The blue atom is nitrogen-atom. (**c**) The big opening of binding pocket to the solvent that consists of α1, α3, α4. (**d**) The small opening of binding pocket to the solvent that consists of α4, α5.

To assess ligand binding, we docked 58 volatiles and 1-NPN into the BhorOBPm2 binding pocket using Surflex-Dock in Sybyl-X. BhorOBPm2 has four cysteine residues and belongs to the minus-C insect OBP family (Fig. 3). After removal of the signal peptide, a total of two disulfide bridges were observed between Cys22 and Cys53, and Cys91 and Cys111. The positions of those cysteine residues were similar to Cys26 and Cys57, and Cys95 and Cys113 of 2ERB. However, 2ERB has a third disulfide bridge between Cys53 and Cys104. Based on data from ESPript3.0 (http://espript.ibcp.fr/ESPript/ESPript/) and MOE 2012, the BhorOBPm2 binding pocket is formed by six α-helices, with residues 11 through 26 in α1, 31 through 38 in α2, 45 through 57 in α3, 69 through 73 in α4, 78 through 88 in α5, and 102 through 113 in α6 (Fig. 3a). Similar to AmelOBP1, BhorOBPm2 has a C-terminal region that forms a wall over the binding pocket adjacent to the N-terminal α-helix (Fig. 3b). The carboxylate of the C-terminal Phe123 formed a hydrogen bond with Tyr50 (Fig. 3c). The docking result of all ligands showed a tunnel formed in the BhorOBPm2 core (Fig. 4a), with two solvent-exposed openings consisting of α1, α3, and α4 (Fig. 4c), and the smaller opening formed by α4 and α5 (Fig. 4d). Notably, most regions of the binding pocket were hydrophobic (Fig. 4a), except for the C-terminal wall (Fig. 4b). Residues Ile121 and Phe123 in the C-terminal region formed the only polar surfaces in the pocket, with side-chain and backbone oxygen atoms oriented toward the center of the pocket (Fig. 4b).

### pH-dependent binding and the pH-sensitive C-terminal region

**Figure 5:**
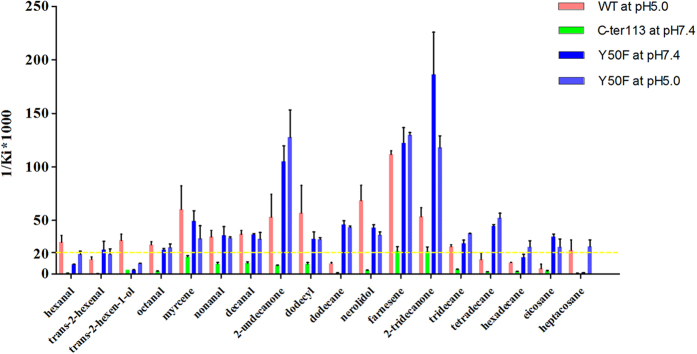
Comparison of binding affinities (indicated by 1/Ki*1000) between BhorOBPm2 and its mutant C-ter113, Y50F at different pH to ligands with long chain.

As seen in Supplementary Table S2, wild-type BhorOBPm2 bound ligands with significantly lower affinities at pH 5.0 as compared to those observed at pH 7.4. This was consistent with findings associated with AaegOBP1. AageOBP1 studies speculated that the C-terminal region acted as a pH-sensitive “lid” that could be opened following disruption of any array of acid-labile hydrogen bonds^15^. To test this finding on BhorOBPm2, we mutated the C-terminus to simulate the open state of the C-terminal region. We observed that the mutant protein C-ter113 exhibited ligand-binding affinities at pH 7.4 different from those observed in wild-type BhorOBPm2 at pH 5.0. Wild-type BhorOBPm2 exhibited binding affinities of 1/K_i_(μM) ×1000 > 20 (K_i_ < 50 μM) at pH 5.0, which were lower than those observed at pH 7.4; however, the mutant protein C-ter113 exhibited no ability to bind ligands at pH 7.4 (1/K_i_ (μM) × 1000 < 20 (K_i_ > 50 μM)) (Fig. 5). The lack of a “lid” blocking the binding pocket may result in ligands being unable to maintain their position in the pocket. Moreover, elimination of the hydrogen-bond between Tyr50 and Phe123 may have also affected this region. The mutant protein Y50F exhibited binding affinities with ligands and the order of 1/K_i_(μM) × 1000 > 20 (K_i_ < 50 μM) at pH7.4, which was similar to those observed for wild-type BhorOBPm2 at pH 5.0. Notably, the pH did not significantly influence binding affinities in the mutant variant (Fig. 5).

## Discussion

Here, we identified the minus-C OBP BhorOBPm2 that formed two disulfide bridges according to the predicted 3D structure. We also quantified BhorOBPm2 binding affinities in the presence of different ligands.

**Figure 6:**
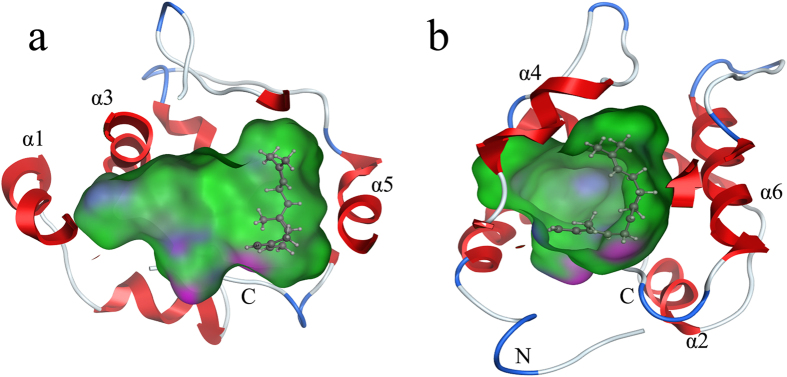
Docking result of BhorOBPm2 with farnesene. The gray molecule in the pocket is farnesene. The green areas express hydrophobicity and red areas express hydrophilia of binding pocket. (**a**) Farnesene was bound to one side of the pocket in hydrophobic areas. (**b**) Farnesene was bent and folded in the pocket.

We obtained optimal positioning for all ligands in the BhorOBPm2 binding pocket, which allowed observation of the binding pocket taking on an elbow-like shape (Fig. 4a). The interactions between ligand and binding pocket contribute to binding affinity; however, we noted that the ligand farnesene was bound to one side of the pocket instead of occupying the entire area due to the hydrophobicity of the pocket (Fig. 6a). Other ligands displaying adequate binding affinity also showed different positioning in the pocket that appeared to primarily depend upon hydrophobic interactions (Supplementary Fig. S5). Once bound, all ligands left unfilled areas in the binding pocket. Ligands with longer chains, such as pentacosane, exhibited low binding affinities, although they appeared to be more suitable for the shape of pocket (Supplementary Fig. S5). We speculated that the pocket may have sufficient capacity to bind two or more short-chain ligands, which was in agreement with findings from studies of CSPMbraA6^35^. In the case of our experiments, this binding pattern would lead to competitive binding between the ligand and 1-NPN, resulting in an N:1 stoichiometry instead of 1:1, thereby lowering fluorescence intensity. Our simulations resulted in lower affinities relative to those obtained from experimental data. As the chain length increased, competitive binding between the ligand and 1-NPN resulted in a 1:1 stoichiometry, enabling the binding affinities to increase. Binding affinities depend upon the shape of ligands and their suitability for the binding pocket. When the chain length exceeds that of the binding pocket, increased collision events between ligand and pocket could occur, thereby influencing the binding affinity. Additionally, in the case of circular ligands, another important aspect is to consider their weaker elasticity as compared with ligands containing carbon chains. A certain degree of conformational flexibility might allow ligands to access the central binding pocket in OBPs, particularly those of LUSH^17^,^36^, AgamOBP4^37^, and AmelASP2^38^. The ligands with carbon chains could be capable of conformational changes enabling their access to the binding pocket, enabling a given protein to bind a range of ligands having carbon chains due to their elasticity^24^.

Various structures of insect OBPs have been reported, but studies of crystal structure are still fewer. Among these crystal structures, we found that BhorOBPm2 exhibited similar structure and binding characteristics with one type of classical OBPs, including AgamOBP1, CquiOBP1 and AaegOBP1. BhorOBPm2 showed the highest degree of sequence similarity with AgamOBP1 which revealed a continuous channel through the dimeric interface. Comparing with AgamOBP1 which could accommodate a chain of at least 40 PEG atoms, BhorOBPm2 showed low binding affinities with excessive carbon chains, such as pentacosane and heptacosane, although the BhorOBPm2 binding pocket exhibited an elbow-like shape similar to that of AgamOBP1 (Fig. 4a). In AgamOBP1, the channel from the “elbow” of the channel to the solvent surface would be unoccupied upon ligand binding, only a part of the ligand molecule was binding in the central pocket. While ligand docking showed that the ligand farnesene was able to fold itself in one side of the BhorOBPm2 pocket (Fig. 6b). It seemed that BhorOBPm2 left larger space for ligands binding compared with AgamOBP1. Notably, CquiOBP1 exhibited high binding affinities with octanal, nonanal, and decanal, which agreed with our findings for BhorOBPm2. However, octanal showed apparent higher affinity with CquiOBP1 as compared with others, implying that the shorter hydrophobic chain fit better in the CquiOBP1 binding pocket. In the case of BhorOBPm2, farnesene exhibited the highest binding affinity, while other ligands with longer carbon chains, including 2-tridecanone, tridecane, and tetradecane also showed higher affinities as compared with that of octanal. These findings suggested that BhorOBPm2 has a larger binding pocket as compared with that of CquiOBP1 in order to accommodate ligands having longer carbon chains.

The only crystal structure of minus-C OBPs was that done for *A. mellifera* (Amel) OBP14^20^. BLAST analysis showed 15.5% identity with BhorOBPm2, although BhorOBPm2 also had four cysteines. The structures differed in their C-terminal region, which formed helix 7 in AmelOBP14^20^. However, as the most representative characteristic of the minus-C OBP family, the positions of the two disulfide bridges in BhorOBPm2 were similar to those between Cys17 and Cys49, and Cys88 and Cys106 in AmelOBP14 (Supplementary Fig. S4). Additionally, the different shapes in the binding pockets revealed a closed core in AmelOBP14 relative to an open pocket in BhorOBPm2, which determines the differences in ligand selection. Elongated compounds may, therefore, be better suited to BhorOBPm2, while bulky, cyclic compounds with hydrogen-bond acceptor might be favorable to AmelOBP14^20^. Given the CquiOBP1 was compared to a “broadband filter”^14^, BhorOBPm2 was also capable of acquiring ligands selected based on the length of their hydrophobic chain. While the plasticity and volume of the binding pocket may help AmelOBP14 bind a range of candidate odorant molecules^20^, the selective specificity of ligands to pocket volume was also reported in minus-C OBP DhelOBP21^24^. These findings supported that, although BhorOBPm2 and AmelOBP14 sequence similarities enabling the formation of two disulfide bridges, their binding mechanism varied considerably.

AmelOBP14 studies postulated that minus-C OBPs might be ancestral proteins from which classical OBPs have evolved^20^. Subsequent studies of mutant AmelOBP14 variants containing a third disulfide bond showed lower odorant affinity. One possible explanation for this is the constricted flexibility introduced by the additional disulfide bond^21^. We also observed the influence of disulfide bonds in our study of BhorOBPm2, which has a larger binding pocket that allows it broader ligand specificity as compared with classic OBPs AgamOBP1 and CquiOBP1, despite their having similar structures and binding capabilities involving ligands with carbon chains. It appeared that the increased flexibility due to the lack of an extra disulfide bond, which contributed to the observed difference. These findings suggested that BhorOBPm2 exhibited an intermediate structure between minus-C OBPs and some classic OBPs including AgamOBP1, CquiOBP1 and AaegOBP1, implied that this minus-C OBP might be ancestral proteins of these classic OBPs.

**Figure 7:**
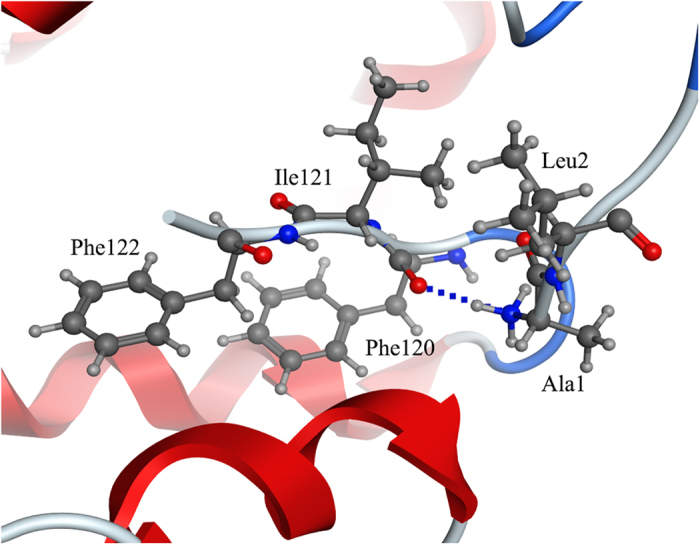
Predicted hydrogen bond in the C terminus. The related residues have been identified (black numbers). The red atom is oxygen atom. The blue atom is nitrogen-atom. Hydrogen bond is shown as blue dotted lines.

Many studies showed that OBPs underwent pH-dependent conformation changes that lowered ligand affinity. AaegOBP1 studies described the C-terminus as a “lid” that could be opened following disruption of an array of acid-labile hydrogen bonds as an explanation of the reduced binding affinity at low pH^15^,^39^. However, the binding affinities reported here for the mutant BhorOBPm2 variants were inconsistent with findings. The mutant protein C-ter113 resulted in simulation of the open state and exhibited no ligand-binding ability at pH 7.4 (K_i_ > 50 μM), while wild-type BhorOBPm2 exhibited binding affinities of K_i_ < 50 μM at pH 5.0. Truncating the C-terminal region caused more significant alterations to the protein structure as compared with the change in pH. Moreover, the mutant protein Y50F, which eliminated the hydrogen-bond between Phe123 and Tyr50, exhibited similar ligand-binding affinities at pH 5.0 and 7.4 as those observed for the wild-type variant at pH 5.0. If the open state of the C-terminal region was required to disrupt an array of acid-labile hydrogen bonds, it was unclear why the Y50F mutant variant would exhibit similar binding affinity at pH 7.4 as that of the wild-type variant at pH 5.0, or why the Y50F mutant did not exhibit a lower ligand-binding affinity at pH 5.0 relative that observed at pH7.4. Tyr122, Phe123, and Leu124 in AaegOBP1 form hydrogen bonds with the N**-**terminus and α-helix 2. The subsequent conformation reportedly changed and following hydrogen-bond disruption at pH 5.0^39^. However, in the same position in BhorOBPm2, Ile121 and Phe122 did not form hydrogen bonds with other residues, and Phe120 only formed one hydrogen bond with Ala1 (Fig. 7). Furthermore, with hydrophobic Leu2 in BhorOBPm2 was in the place of the polar residue Arg6 in AaegOBP1. It appeared that the C-terminal region of AaegOBP1 exhibited a weaker link with the N-terminal region and α-helix 2. Therefore, this finding may be suitable as a comparative example for our findings related to BhorOBPm2. Our hypothesis considered that the C-terminal region might move toward the binding pocket following breakage of the hydrogen bond between Phe123 and Tyr50, resulted in the pocket allowing the ligands to escape from the pocket though the pocket opening. Specifically, the polar elements in the pocket wall formed by the C-terminus were unfavorable for binding hydrophobic ligands and may have contributed to their exclusion during the release process. Similar conditions wherein the C-terminal region occupied the ligand-binding site were reported in a study of BmorPBP and a PBP from the giant silk moth *A. polyphemus*^40^. However, the C-terminal tail of BmorPBP was long enough to form a helix that could fit into the binding pocket^40^. While the C-terminal region of BhorOBPm2 was not as long as that of BmorPBP, it was still capable of forming a wall over the binding pocket and acting similar to a piston that pushed ligands out of the pocket at pH 5.0, thereby opening the pocket for binding at pH 7.4.

Our study suggests that BhorOBPm2 specifically recognizes the ligands based on chain length, which provides a useful basis on the molecular level to find a convenient substitute ligand. In future studies, we will focus on the effects of the selected ligands on the behavior and ecology of *B. horsfieldi* for longhorned beetle control. Furthermore, there are many OBPs in nature, each exhibiting different mechanisms for ligand binding and releasing. Further research is required to elucidate the relationships between sequential evolution and structural features, which will enable systematic understanding of OBP function.

## Materials and Methods

### Insects

*B. horsfieldi* adults were collected in Gong’an County of Hubei Province in China (112°23′E; 30°04′N). *B. horsfieldi* used for RNA extraction were reared in plastic containers with fresh twigs from *R. multiflora*.

### Chemicals

Compounds used in the binding assays (58) were purchased from Sigma-Aldrich (St. Louis, MO, USA) and stored according to manufacturer instructions.

### RNA extraction and cDNA synthesis

Total RNA was extracted from the antennae of *B. horsfieldi* using Trizol reagent (Invitrogen, Carlsbad, CA, USA) according to manufacturer instructions. RNA concentration was determined by ultraviolet absorbance in a BioPhotometer Plus spectrophotometer (Eppendorf, Hamburg, Germany). A reverse-transcription polymerase chain reaction (PCR) system (Promega, Beijing, China) was used to reverse transcribe the isolated RNA into cDNA.

### Recombinant-plasmid construction

The sequence encoding BhorOBPm2 was amplified by PCR with a forward primer containing an *EcoR*I-restriction site (5′-CCGGAATTCATGGATAGCTTAATATTTCTAG-3′) and a reverse primer containing a *Xho*I-restriction site (5′-CCGCTCGAGTTAGAAGAAAATAAATGTTTC-3′). The PCR product was ligated into a pMD-18T vector using a 1:5 (plasmid:insert) mass ratio, and the ligation product was transformed into DH5α *Escherichia coli* competent cells. After identification by PCR, the positive clone was selected and grown in Luria-Bertani (LB) medium with kanamycin (50 μg/ml) and then sequenced. Target fragments digested from the pMD-18T recombinant plasmid were ligated into a pET-30a plasmid, and the recombinant plasmid was transformed into DH5α *E. coli* competent cells. After DNA sequencing, BL21 (DE3) *E. coli* cells were transformed with the correct recombinant plasmid. A single clone was grown in LB medium containing kanamycin (50 μg/ml) with shaking overnight at 220 rpm and 37 °C, followed by sequencing.

### Recombinant-protein expression and purification

The positive clone verified by DNA sequencing was inoculated in 5 mL LB medium with kanamycin (50 μg/mL) with shaking at 220 rpm at 37 °C. After 4 to 6 h, the culture was diluted to 1 L LB medium and grown to an OD_600_ of 0.4 to 0.6, after which 0.1 mM isopropyl-beta D-thiogalactopyranoside (IPTG) was added, followed by culturing for 4 h at 37 °C. The expressed protein presented as inclusion bodies was solubilized by addition of 10 mL 8 M urea in 50 mM Tris buffer (pH 7.4) and incubated in 1 mM dithiothreitol. We then added 200 μL of 100 mM cysteine in 0.5 M NaOH and 15 mL of 5 mM cysteine in 100 mM Tris buffer (pH 8.0). Before purification, the protein solution was dialyzed eight times every 2 h into 30 mM Tris buffer (pH 7.4). The recombinant protein was purified by an affinity Ni-chromatography column (GE Healthcare, Uppsala, Sweden). Recombinant bovine enterokinase was added to the eluted proteins and incubated at 26 °C for 10 h to remove the His-tags from the recombinant proteins. Protein expression and purification was assessed by 15% sodium dodecyl sulfate polyacrylamide gel electrophoresis (SDS-PAGE). The purified protein was dialyzed in Tris buffer (pH 7.4) and (pH 5.0), and the concentration was determined prior to performing fluorescence-binding assays. The purified proteins were stored at −80 °C until use.

### Fluorescence-binding assays

Fluorescence competition-binding assays were performed to determine the binding affinity of the BhorOBPm2 protein for various ligands using 1-NPN as a fluorescent probe. The binding affinity for 1-NPN was determined by adding aliquots of 1 mM 1-NPN into a 2-μM protein sample in 30 mM Tris-HCl to final concentrations of 0 μM to 28 μM at room temperature. The fluorescence of 1-NPN was excited at 337 nm, and emission was recorded at between 350 nm and 600 nm using a RF-5301PC fluorescence spectrophotometer (Shimadzu, Kyoto, Japan), a 1-cm light path, and a quartz cuvette. The competitive binding of ligands was measured using 1-NPN (2 μM) as the fluorescent probe with a stoichiometry of 1:1 (protein:ligand), with the final concentration of each ligand added to the sample ranging from 0 μM to 20 μM. Reduction in relative fluorescence intensity indicated that the competitor displaced 1-NPN from the binding site of BhorOBPm2. The binding data were obtained from three independent measurements. The dissociation constants (*K*_*d*_) of the OBPs for 1-NPN were calculated from Scatchard plots of the binding data using the Prism 5 software (GraphPad, La Jolla, CA, USA). Binding affinities (K_i_) of the OBPs for each ligand were determined based on the IC_50_ value (the initial fluorescence level of the competitor concentration reduced by half) using the equation: K_i_ = [IC_50_]/(1 + [1-NPN]/K_1-NPN_), where [1-NPN] is the free concentration of 1-NPN, and K_1-NPN_ is the dissociation constant of the complex BhorOBPm2/1-NPN.

### Molecular modeling and ligand docking

Delta–BLAST (http://blast.ncbi.nlm.nih.gov/Blast.cgi?PAGE_TYPE=BlastSearch&PROGRAM=blastp&BLAST_PROGRAMS=deltaBlast) was performed with the BhorOBPm2 sequence against the Protein Data Bank (PDB; http://www.rcsb.org) on the SWISS-MODEL server (SWISS-MODEL; http://swissmodel.expasy.org/). Sequence identities >30% were chosen for subsequent analysis.

BLAST results were subjected to ClustalW2 analysis to obtain a multiple-sequence alignment and phylogram. Molecular Operating Environment (MOE) version 2012.10^41^ was used for homology modeling as follows: 1) the homologous-protein profile was first aligned according to sequence similarity and secondary structure; 2) BhorOBPm2 was then aligned using that alignment; 3) the best protein was selected based on homology, evolution, sequence similarity, the number of cysteines, the phylogram, and several techniques for structure determination, and this template was used to build a 3D model of BhorOBPm2. In the modeling procedure, we set the maximum number of main-chain models to 50 and the side-chain samples at 300 K to five. For model refinement, “intermediates” and the “final model” parameters were set to “fine”, and AMBER99 was selected as the force field, while other parameters were set to the defaults.

After obtaining the model, it was subjected to sufficient stereochemical refinement and energy minimization according to the electrostatic solvation energy, which was calculated using the generalized Born/volume integral method. Further refinement was performed based on Protonate 3D in MOE. Notably, in molecular docking, reproduction of the complex crystal structure is both a necessary prerequisite and a challenging issue. Protonate 3D is a powerful tool that can assign ionization states and position hydrogens in a macromolecular structure based on their 3D coordinates (typically from a crystal structure).

Subsequently, the stereochemical structure of the model was checked in MOE, involving dihedral angles (ψ and φ), bond lengths, bond angles, rotamers, and atom clashes. The best BhorOBPm2 model with the lowest electrostatic solvation energy and optimal geometric properties were selected for follow-up molecular-docking analysis. After the tertiary structure was obtained, 56 volatiles and 1-NPN were docked into the BhorOBPm2 pocket, which was established by employing the Surflex-Dock suite embedded in Sybyl-X version 2.0^42^.

In the docking suite, Surflex-Dock was selected as the docking mode, and a multi-channel surface was set as the protomol-generation mode. Subsequently, “bloat” was set to 2 Å, the additional starting conformations per molecule were increased to 10, the density of search was set to six, and the “consider ring flexibility” parameter was checked. All of these parameters were set to improve docking accuracy. Finally, minimum root-mean-square deviation (RMSD) between final poses was set to 0.5 Å to allow exploration of additional docking poses and to achieve higher accuracy. Other parameters were set to default values. Following the docking process, the ligand-binding patterns with the OBP receptor were explored and identified, and the mechanisms of interaction between signal molecules and BhorOBPm2 were analyzed.

On the basis of the docking results, two mutants were used to verify the function of the C-terminal region: C-ter113 (removal of the C-terminal region from His113) allowed simulation of the C-terminal region being “open”, whereas incorporation of a Y50F (tyrosine to phenylalanine) mutation allowed transformation of the C-terminal region by eliminating hydrogen-bond interaction between Tyr50 and Phe123, while retaining other hydrogen-bonds. The mutant proteins were re-docked with the volatiles according to the same method described.

### Site-directed mutagenesis

The BhorOBPm2 protein was mutated to two mutants: Y50F and C-ter113. The Y50F mutant was generated using the Fast Mutagenesis System (TransGen, Beijing, China) according to manufacturer protocol, using the recombinant plasmid pET30a-OBPm2 as the template. The C-ter113 mutant was generated by PCR with specific primers, with the products ligated into the pET-30a plasmid. The correct mutations were verified by DNA sequencing, and expression and purification of the mutant proteins were performed as described previously.

## Additional Information

**How to cite this article**: Zheng, Z.-C. *et al*. Predicted structure of a Minus-C OBP from *Batocera horsfieldi* (Hope) suggests an intermediate structure in evolution of OBPs. *Sci. Rep.* **6**, 33981; doi: 10.1038/srep33981 (2016).

## Supplementary Material

Supplementary Information
