# Supplementary material for: Predicted structure of a Minus-C OBP from Batocera horsfieldi (Hope) suggests an intermediate structure in evolution of OBPs
Source: Sci Rep. 2016 Sep 23;6:33981. doi: 10.1038/srep33981 (PMC5034290; doi:10.1038/srep33981)
Supplement: Supplementary Information [file srep33981-s1.pdf]

Supplementary Information

Predicted structure of a Minus-C OBP from *Batocera horsfieldi* (Hope) suggests an intermediate structure in evolution of OBPs

Zhi-Chuan Zheng[#], Dong-Zhen Li[#], Aiming Zhou, Shan-Cheng Yi, Hao Liu, Man-Qun Wang*

Supplementary Table S1. List of compounds tested for binding assays.

	CAS	Ligands	length of chain		CAS	Ligands
	584-02-1	3-pentanol	5		96-17-3	2-methylbutyraldehyde
	1576-95-0	cis-2-penten-1-ol	6		616-25-1	1-penten-3-ol
	110-54-3	hexane	6		141-78-6	ethylacetate
	66-25-1	hexanal	7		108-93-0	cyclohexanol
	6728-26-3	trans-2-hexenal	7		90-02-8	salicylaldehyde
	928-95-0	trans-2-hexen-1-ol	7		99-87-6	4-lsopropylto luene
	543-49-7	2-heptanol	7		93-58-3	methyl benzoate
	928-96-1	cis-3-hexen-1-ol	7		79-92-5	camphene
	111-65-9	octane	8		99-83-2	α-phellandreene
	124-13-0	octanal	8		99-86-5	α-terpinene
	3391-86-4	1-octen-3-ol	8		13466-78-9	(+)-3-carene
	123-35-3	myrcene	9		7785-70-8	(R)-(+)-a-pinene
	928-95-0	E-2-hexen-1-ol acetate	9		5989-54-8	（-）-limoonene
	124-19-6	nonanal	9		5989-27-5	R-(+)-limonene
GROUP 1	112-31-2	decanal	10	GROUP 2	214-807-2	(S)-(-)-verbenone
	112-12-9	2-undecanone	11		76-22-2	（+/—）-camphor
	112-54-9	dodecyl aldehyde	12		1195-92-2	（-）-Limonene oxide
	112-40-3	dodecane	12		4695-62-9	(+)-Fenchone
	7212-44-4	nerolidol	12		20126-76-5	(-)-terpinen-4-ol
	502-61-4	farnesene	12		546-79-2	sabinene hydrate
	593-08-8	2-tridecanone	13		10482-56-1	α-Terpineol
	629-50-5	tridecane	13		124-76-5	isoborneol
	629-59-4	tetradecane	14		469-61-4	（-）-α-cedrene
	544-76-3	hexadecane	16		1135-66-6	(-)-isolongifolene
	593-45-3	octadecane	18		1137-12-8	（+）-longicyclene
	629-92-5	nonadecane	19		5989-08-2	(+)-α-longipinene
	112-95-8	eicosane	20		77-53-2	(+)-cedrol
	593-49-7	heptacosane	21			
	629-99-2	pentacosane	25			

Supplementary Table S2. Binding data (indicated by 1/Ki(uM)*1000) of the BhorOBPm2 and its mutant with different ligands.

	WT		C-ter113		Y50F	
ligands	pH7.4	pH5.0	pH7.4	pH5.0	pH7.4	pH5.0
3-pentanol	13.60	62.92	0.40	2.89	5.58	0.57
cis-2-penten-1-ol	28.91	3.18	10.02	19.33	24.00	11.06
hexane	2.31	15.26	3.21	5.20	0.30	27.12
hexanal	52.25	29.94	0.63	1.60	9.00	18.46
trans-2-hexenal	37.92	13.67	0.42	0.91	22.81	18.69
trans-2-hexen-1-ol	37.38	31.53	3.69	8.43	3.31	9.85
2-heptanol	3.31	11.35	1.08	4.62	9.09	16.28
cis-3-hexen-1-ol	13.18	18.70	2.20	8.55	28.90	12.59
Octane	13.73	11.15	2.58	2.97	19.27	2.80
Octanal	54.09	27.48	2.63	1.21	22.90	24.74
1-octen-3-ol	25.60	2.35	0.50	9.08	4.80	4.18
myrcene	77.14	60.49	16.25	3.04	49.41	32.96
E-2-Hexen-1-ol acetate	16.97	2.38	0.10	11.01	13.25	33.00
nonanal	50.40	34.65	9.42	13.36	35.94	33.69
decanal	102.21	37.12	10.12	9.53	36.92	32.50
2-undecanone	42.02	53.17	7.77	0.26	105.10	127.70
dodecyl aldehyde	119.74	57.01	9.26	12.79	32.61	32.40
dodecane	216.98	9.93	1.04	0.55	45.88	43.74
nerolidol	220.31	68.75	3.25	3.12	43.05	36.39
farnesene	1159.11	111.78	21.65	31.93	122.10	129.99
2-tridecanone	353.05	53.55	21.20	14.34	186.20	117.95
tridecane	262.09	25.76	4.26	8.52	28.60	37.70
tetradecane	94.91	13.38	1.76	18.24	44.61	52.29
hexadecane	67.53	10.82	2.37	1.51	15.54	25.39
octadecane	21.69	2.33	6.23	2.09	35.76	26.76
nonadecane	4.63	11.38	4.42	3.01	8.06	30.51
eicosane	42.12	5.08	2.63	9.05	34.80	25.39
heptacosane	45.56	22.35	0.53	11.80	1.22	25.60
pentacosane	1.89	0.09	3.73	7.63	12.29	11.39
2-methylbutyraldehyde	28.10	28.11	1.65	7.64	39.37	10.27
1-penten-3-ol	4.35	4.71	6.03	19.10	3.68	56.01
ethylacetate	2.60	21.98	0.02	1.25	23.61	20.89
cyclohexanol	16.11	8.56	1.62	9.43	2.12	20.82
salicylaldehyde	0.90	2.17	3.31	0.89	0.73	10.56
4-lsopropylto luene	18.01	8.67	0.01	3.69	3.62	6.59
methyl benzoate	17.19	9.13	2.26	14.60	3.27	9.55
camphene	22.05	1.38	10.49	4.24	0.31	6.41

α-phellandreene	19.19	3.83	2.18	18.00	14.07	5.72
a-terpinene	8.67	11.87	7.33	0.53	1.68	12.56
(+)-3-carene	10.97	10.02	1.32	11.43	17.64	44.87
(R)-(+)-a-pinene	14.71	14.78	0.82	1.73	5.83	10.46
（-）-limoonene	8.12	9.58	0.02	14.24	5.12	13.29
R-(+)-limonene	7.42	46.10	0.26	10.22	1.47	5.06
(S)-(-)-verbenone	4.57	2.92	3.06	0.24	14.79	20.62
（+/—）-camphor	2.66	0.37	0.72	6.99	21.38	28.56
（-）-limonene oxide	11.38	13.44	15.53	21.41	4.41	9.78
(+)-fenchone	6.16	3.92	0.14	3.05	33.38	1.49
(-)-terpinen-4-ol	37.35	5.94	1.97	8.50	25.96	13.30
sabinene hydrate	23.71	43.81	1.64	0.14	11.70	6.43
α-terpineol	4.65	8.95	16.05	11.01	13.08	9.00
isoborneol	16.47	4.25	5.59	0.78	0.99	18.22
（-）-α-cedrene	24.50	69.60	0.37	11.80	34.37	60.36
(-)-isolongifolene	46.93	11.08	28.12	21.69	30.90	25.43
（+）-longicyclene	16.06	2.93	0.31	1.21	23.09	12.82
(+)-α-longipinene	63.19	38.53	11.23	10.22	47.71	78.17
(+)-cedrol	18.57	0.86	0.02	0.24	6.36	24.35

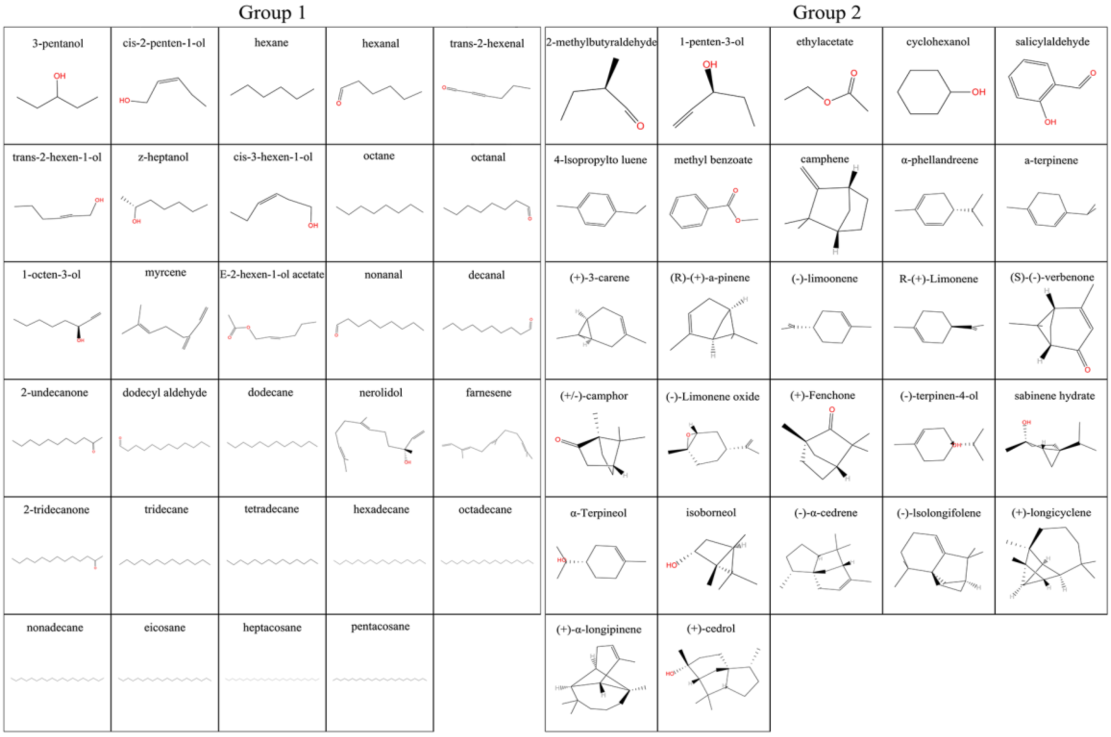

Supplementary Figure S1. Plane structure and classification of ligands. (A) ligands with long chain. **(B)** ligands without long chain.

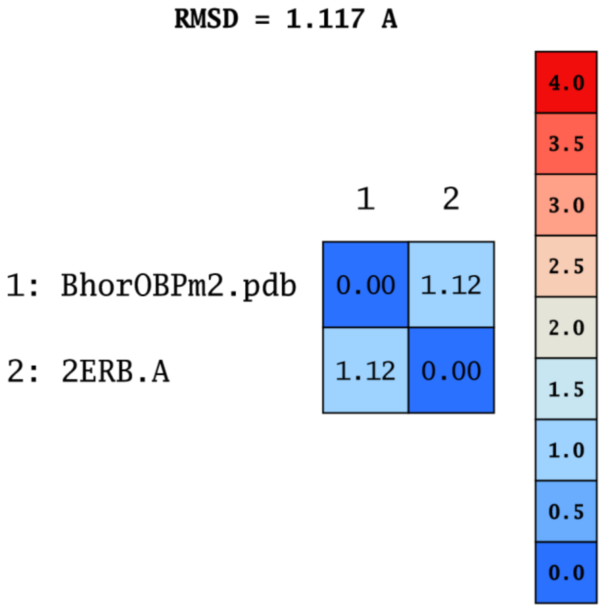

Supplementary Figure S2. A pairwise RMSD of alpha C between the template 2ERB and BhorOBPm2.

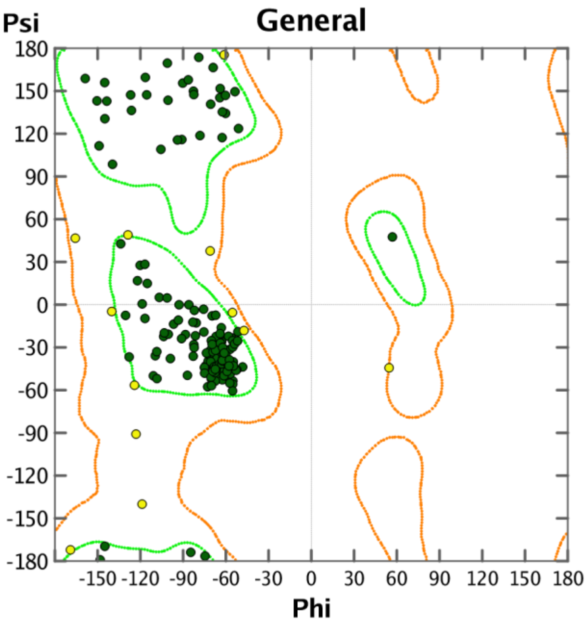

Supplementary Figure S3. The Ramachandran map of the model of BhorOBPm2. The scope of orange dashed line expresses the allowed region. The scope of green dashed line expresses the core region. The yellow square means the amino acid located within the allowed region. The green square means the amino acid located within the core region.

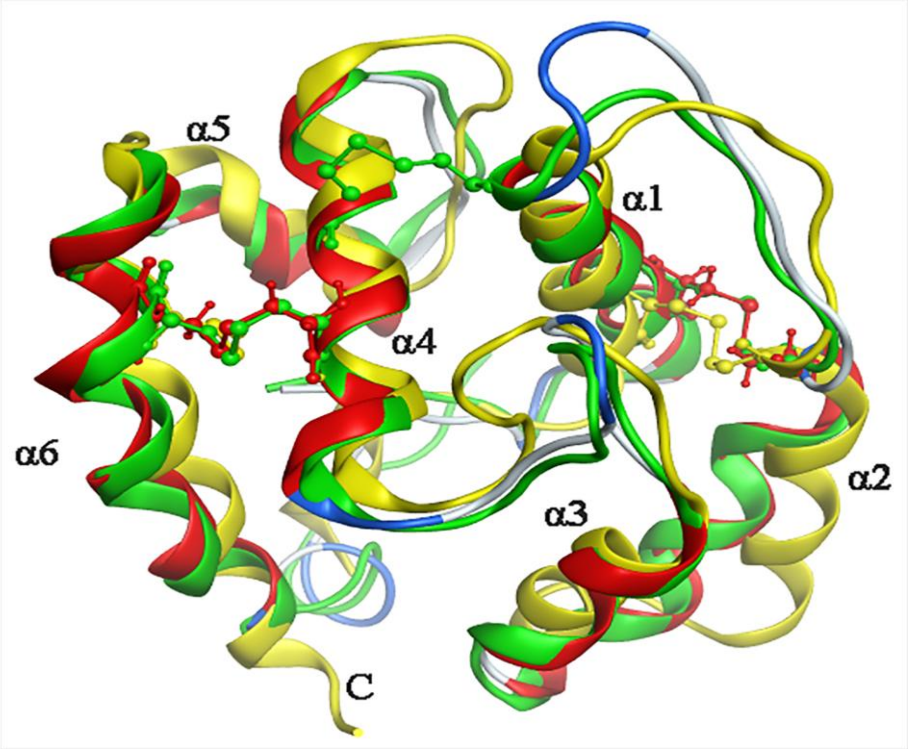

Supplementary Figure S4. Superimposed structures of BhorOBPm2, 2ERB and AmelOBP14. The model of BhorOBPm2 and crystal structure of 2ERB and AmelOBP14 are shown in red, green and yellow, respectively. The Cys residues forming the disulfide bridges were identified as stick models.

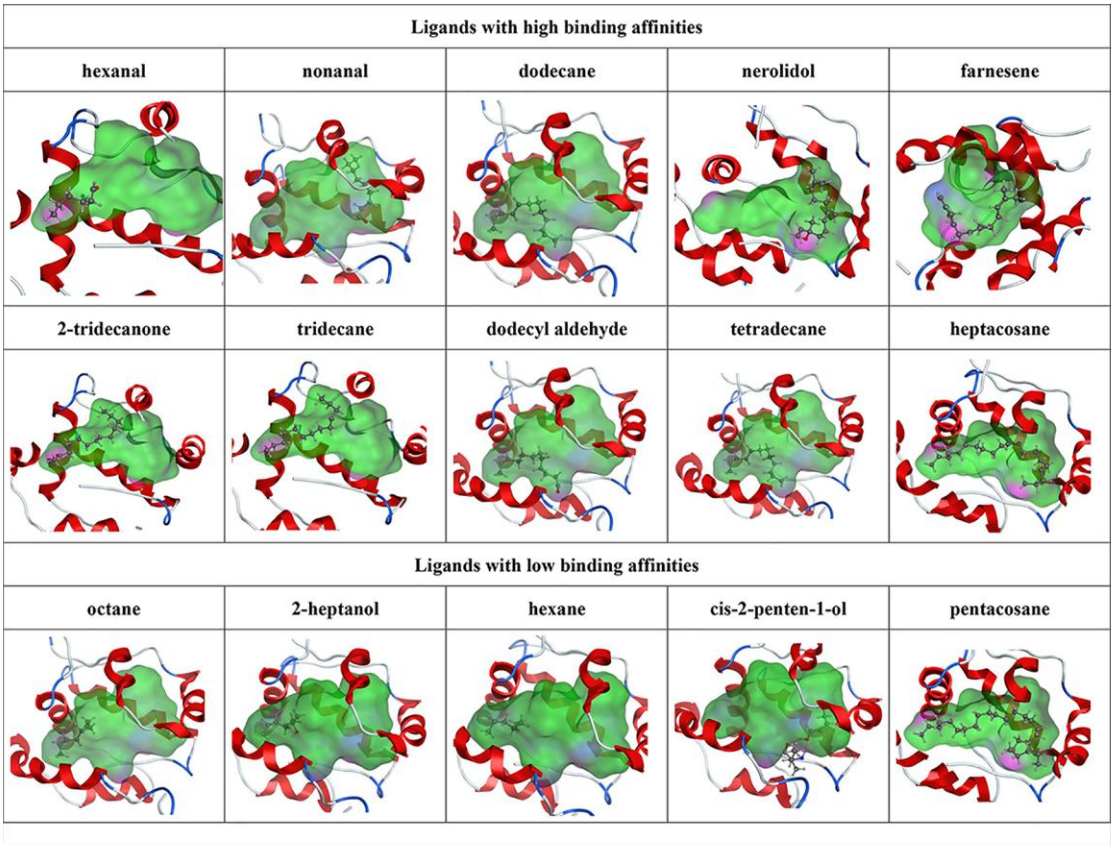

Supplementary Figure S5. Docking results of BhorOBPm2 with the long chain ligands. The green areas express hydrophobicity and red areas express hydrophilia of binding cavity. The gray molecule in the cavity is ligand.

Ligands with high binding affinities		
(+)-α-longipinene	(-)-isolongifolene	(-)-terpinen-4-ol
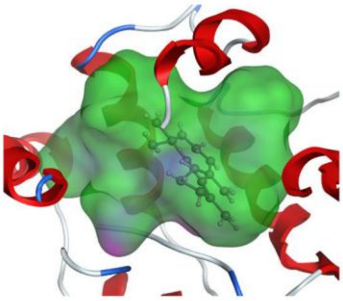	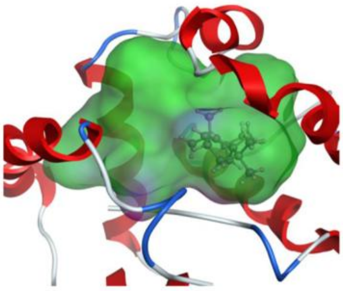	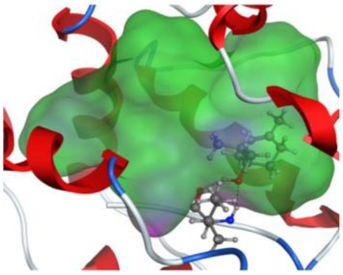
Ligands with low binding affinities		
(S)-(-)-verbenone	α -terpinene	sabinene hydrate
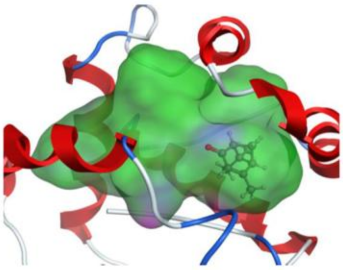	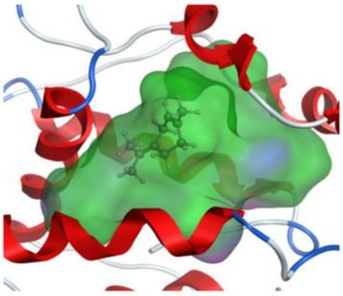	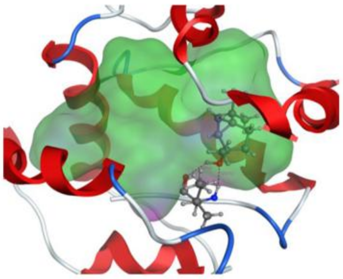

Supplementary Figure S6. Docking results of BhorOBPm2 with the circular ligands. The green areas express hydrophobicity and red areas express hydrophilia of binding cavity. The gray molecule in the cavity is ligand.
